# Evaluation of High-Temperature Sterilization Processes: Their Influence on the Mechanical Integrity of Additively Manufactured Polymeric Biomaterials

**DOI:** 10.3390/ma18061356

**Published:** 2025-03-19

**Authors:** Barbara Zbyrad, Małgorzata Zaborniak, Łukasz Kochmański, Katarzyna Jasik, Janusz Kluczyński, Grzegorz Budzik, Paweł Turek

**Affiliations:** 1 Faculty of Mechanical Engineering and Aeronautics, Rzeszow University of Technology, 35-959 Rzeszow, Poland; zbyrad13@gmail.com (B.Z.); mzab@prz.edu.pl (M.Z.); l.kochmanski@prz.edu.pl (Ł.K.); gbudzik@prz.edu.pl (G.B.); pturek@prz.edu.pl (P.T.); 2 Institute of Robots & Machine Design, Faculty of Mechanical Engineering, Military University of Technology, Gen. S. Kaliskiego St., 00-908 Warsaw, Poland; katarzyna.jasik@wat.edu.pl

**Keywords:** biopolymers, additive manufacturing, medical sterilization, strength tests, pelvic implant

## Abstract

The continuous advancement of medical technologies and the increasing demand for high-performance medical devices have driven the search for innovative solutions in biomaterials engineering. However, ensuring the sterility of polymeric biomaterials while maintaining their mechanical integrity remains a significant challenge. This research examines how steam sterilization impacts the mechanical properties of four polymeric biomaterials frequently utilized in medical applications: MED610, PEEK, PET-G HT100, and RGD720. Samples were produced using additive manufacturing (AM), specifically Material Jetting (MJT) and Material Extrusion (MEX) processes, and exposed to steam sterilization at 121 °C and 134 °C. A comprehensive verification process was conducted to ensure the effectiveness of sterilization, including pre-sterilization cleaning, disinfection procedures, and the use of process indicators such as the Bowie–Dick test. Mechanical evaluation included bending tests and Rockwell hardness measurements to assess changes in structural integrity and mechanical strength after sterilization. The results revealed that, while some materials exhibited significant alterations in mechanical properties, others demonstrated high resistance to thermal and humidity exposure during sterilization. These findings provide critical insights into the selection and optimization of polymeric biomaterials for sterilizable medical applications, ensuring their durability and safety in clinical use.

## 1. Introduction

The advancement of medical technologies is closely related to the continuous pursuit of innovative solutions that improve patient care. A major challenge in medical engineering is designing safe and durable biomaterials for use in medical devices and implants. In particular, the growing demand for advanced medical devices has spurred interest in implantology [1,2]. The development of medical implants is a highly complex process that requires extensive research, precision, and meticulous attention to detail at every stage. Over the past few years, additive manufacturing (AM) technologies have gained prominence as effective methods to produce implants [3,4,5,6]. These techniques enable the fabrication of polymeric structures suitable for implantology, offering a high degree of design flexibility. Using prior imaging and computer modeling, AM can create implants with intricate and patient-specific geometries tailored to individual anatomical needs [7,8,9]. Such customized medical solutions present a significant opportunity to improve patient quality of life [10,11,12]. The reputation of additive manufacturing (AM) in the medical sector increased significantly during the COVID-19 pandemic, as this technology was rapidly adopted to manufacture critical healthcare supplies such as personal protective equipment (PPE), ventilator components, and customized medical devices. This demonstrated its flexibility, rapid production capabilities, and ability to address urgent medical needs, further strengthening its value in the healthcare industry [13].

Currently, the most popular AM process families used in medicine include Stereolithography (SLA), various types of Material Extrusion (MEX) technologies, Material Jetting (MJT), Powder Bed Fusion–Laser (PBF-LB) [14,15]. One significant benefit of 3D printing in medicine is its capability to produce patient-specific solutions, improving treatment outcomes and reducing recovery times. Additionally, AM technologies allow for the creation of intricate structures with high precision, which would be challenging or even unattainable with conventional manufacturing methods. This not only enhances the effectiveness of medical implants and prosthetics but also helps in reducing costs and maximizing material efficiency. One of the good examples of the use of the AM technique in medicine is research where the authors [16] used a method based on low-temperature extrusion and lyophilization to develop conductive 3D and 2D structures for bone tissue regeneration aimed at supporting the repair and regeneration of tissues such as the periosteum, nerve membranes, and skin [17,18]. The materials used were polylactic acid (PLA) and polypyrrole (PPy) nanoparticles. The scaffolds developed provided uniform thickness and fine structural stability without defects. Another example is the work published by Bassand et al. [19], who produced drug-controlled release implants made of polylactic-coglycolic acid (PLGA) using hot extrusion. Some other authors [20] developed 3D-printed vascular grafts made of polycaprolactone (PCL) filled with dipyridamole (DIP). The process began with the precise mixing of PCL of different molecular weights with dimethyl iodide (DIP) without the use of solvents, ensuring a homogeneous mixture. The vascular grafts were then printed using AM, allowing control over the geometry and size of the grafts adapted to the specific needs of each patient. Studies confirmed the effective integration of DIP into the PCL matrix and the ability to continuously and controllably release dipyridamole, which is crucial to prevent thrombotic complications and improve biocompatibility. Korelidou et al. [21] presented a solution combining a polylactic acid (PLA)-based rate control membrane with 3D-printed MEX implants to prepare a drug delivery system for implants. This implant can be used postoperatively to ensure localized drug release over an extended period.

Regardless of the manufacturing method, contemporary healthcare increasingly emphasizes the highest quality and effectiveness of patient treatment, which requires strict adherence to safety guidelines and procedures for hospitalized patients [22]. In addition to the medical areas managed by physicians, numerous other fields are developing to support patient health and the work of medical personnel. One key area is sterilization, which is essential for all medical devices, including implants [23,24]. According to experts from the Polish Society for Medical Sterilization, sterilization is the process of destroying reproductive forms of biological pathogens. For an implant to be accepted by the body, it must be completely free of microorganisms. Improper sterilization can lead to infections, pathogen transmission, and even death of the patient. Effective decontamination ensures a Sterility Assurance Level (SAL) of 10^−6^ for medical devices. The decontamination process, which encompasses washing, disinfection, and sterilization, is based on the Spaulding classification, which assesses the risk of infection and assigns appropriate levels of decontamination to medical products. High-risk items (implants and surgical instruments) require sterilization, medium-risk items (flexible endoscopes) may require sterilization or high-level disinfection, and low-risk items (thermometers) require medium or low-level disinfection [25]. Medical sterilization can be performed using various methods, mainly divided into high- and low-temperature processes. Implants, often personalized and single-use, must be meticulously sterilized to avoid changes in physicochemical properties and disease transmission risks. Steam sterilization is one of the most widely used methods due to its effectiveness, ease of control, non-toxicity, and safety [26]. This process requires achieving appropriate temperature and time values, such as 121 °C for 15 min or 134 °C for 3 min. Reserialization steps, such as washing and disinfection, are crucial to reduce biological contamination and ensure effective sterilization [27,28].

Another critical issue is materials that have long supported medicine in both patient treatment and diagnostics. With the advancement of different technologies, the expansion of knowledge of materials science and the suitability of materials in biomedicine, polymeric materials are gaining increasing popularity [29,30,31]. Biomedical materials are subject to ever higher requirements concerning biocompatibility and tolerance by the human immune system, as the introduction of foreign bodies into the organism invariably triggers a defensive response. The intervention of extraneous products can cause inflammatory and allergic conditions and even initiate carcinogenic processes. Implants must not alter the composition of the blood upon contact or affect its clotting mechanism. Biomaterials are biocompatible materials, allowing them to stay in extended contact with body fluids and tissues without triggering harmful reactions, making them suitable for implantology. Polymeric biomaterials can be used in the human body temporarily or permanently. Long-term materials are designed to maintain their mechanical and physicochemical properties for more than 20 years [32,33,34]. 

Biomaterials are fundamental to advancements in modern medicine, enabling the creation of implants, prostheses, and other medical devices that can significantly improve the quality of life. Continuous development of medicine and the constant demand to produce medical devices require the ongoing modernization of engineering technologies. Understanding the effect of sterilization on the characteristics of biomaterials used in implantology is essential to guarantee the safety, durability, and effectiveness of these materials after sterilization, which is crucial for their use in clinical practice. Furthermore, the application of new technologies and materials can improve patient comfort and treatment efficiency, which is significant in the context of the advancement of personalized medicine and individual patient needs. Therefore, this study aimed to examine how steam sterilization affects the selected mechanical properties of four polymeric biomaterials utilized in medical engineering. The analysis sought to evaluate the structural and strength changes in the materials after sterilization processes at different temperatures and under long-term loading. One of the main challenges in sterilization is maintaining the mechanical integrity of polymeric materials, as exposure to high temperatures and humidity can induce structural changes, affecting their performance in medical applications. The choice of sterilization method and process parameters must be carefully optimized within the material process window to ensure both sterility and mechanical stability. This study encompassed the preparation of samples from MED610, PEEK, PET-G HT100, and RGD720 biomaterials using AM technology, conducting steam sterilization processes at 121 °C and 134 °C, and performing mechanical tests such as bend tests and Rockwell hardness tests. The selection of materials was based on their relevance and application in medical engineering. MED610 is widely used for anatomical models and biocompatible surgical guides due to its transparency and mechanical strength. PEEK is known for its excellent thermal stability, high mechanical properties, and biocompatibility, making it a preferred material for permanent implants such as spinal fusion devices. PET-G HT100 was selected due to its improved heat resistance compared to standard PET-G, which ensures better stability under sterilization conditions. Finally, RGD720 was included as a representative photopolymer material commonly used to prototype medical devices, allowing the evaluation of how resin-based AM materials respond to sterilization treatments. These materials were chosen to represent a diverse set of polymeric biomaterials with distinct mechanical and thermal behaviors, allowing for a thorough evaluation of the impact of steam sterilization on AM-manufactured medical components. The structure of this paper is as follows. Section 2 presents the materials and methods used in this study, including AM processes and sterilization protocols. Section 3 discusses the experimental results and their implications for the mechanical properties of the materials. Finally, Section 4 highlights the key findings and suggests possible directions for future research.

## 2. Materials and Methods

### 2.1. Materials for the Research

In this study, four types of polymer materials with potential applications in biomedical engineering were utilized [35,36,37,38]. The first, MED610 is a transparent biocompatible material with high mechanical strength against abrasion, tensile, and flexural forces. It is designed to improve efficiency and cost-effectiveness of manufacturing devices used in medicine and dentistry. The material can maintain prolonged contact with human skin for up to 30 days. However, its contact duration with the mucous membranes is limited to 24 h. This limitation precludes its use as a permanent, nonreplaceable implant. Potential applications and benefits of this material include the creation of anatomical models, patient-specific biocompatible implants, and various medical instruments. MED610 parts can be sterilized using steam, ethylene oxide, or gamma radiation [39,40]. 

The second material used is polyether ketone (PEEK), a thermoplastic, colorless, organic polymer. PEEK is used in the manufacturing of tools, medical equipment, and implants, for example spinal implants, as well as in aerospace, automotive, and related industries. It is characterized by a high melting temperature, reaching approximately 343 °C, making it one of the most thermally stable plastics. PEEK exhibits high mechanical strength, rigidity, and dimensional stability. Its properties, coupled with the fact that it is less heavy than metals, make it an excellent metal substitute. PEEK shows high resistance to cracking and wear and a low coefficient of friction without the need for lubrication. It is biocompatible and suitable for use in contact with the human body. Implants made from this polymer have numerous advantageous properties. They interact easily with the human body, enhancing the effectiveness of devices such as prostheses, and do not cause adverse changes after implantation. Compared to materials that are used more commonly, such as titanium, PEEK implants are lighter and positively impact patient comfort and quality of life. These implants are flexible but maintain their shape under full weight [40].

The next material is polyethylene terephthalate HT100 (PET-G HT100), a specialized variant of PET-G that features improved thermal and mechanical strength and provides greater resistance to extreme working conditions. PET-G HT100 has an increased temperature resistance of up to 100 °C. Combining properties such as dimensional stability, chemical resistance, and high rigidity, AM with PET-G HT100 allows for the production of fully functional components. In the medical field, PET-G HT100 can be used for applications such as the production of anatomical models, laboratory tools, and housings for noninvasive medical devices [31].

The final material is RGD720, a polymer developed by Stratasys Company, which is characterized by high dimensional stability, strength, flexibility, and a smooth surface finish. As a result, it is used in conceptual modeling and transparent devices across various industrial and medical fields, as well as in artistic modeling. RGD720 facilitates visualization and planning of the placement of additional elements in the created product [41].

Table 1 displays the chosen properties of the four materials utilized in this study.

### 2.2. Manufacturing Process

**Figure 1:**
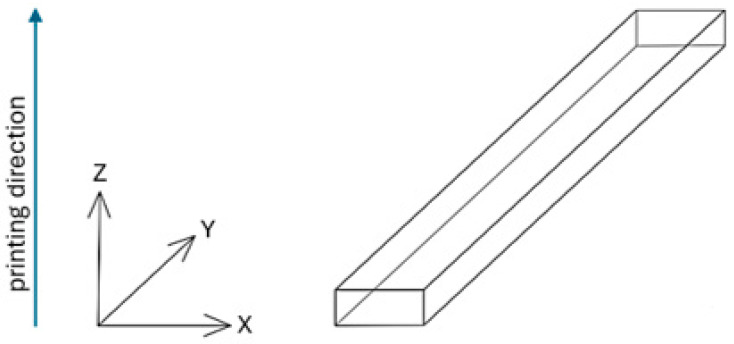
The test samples were oriented along the XY axis during the 3D printing process.

Based on the ISO 1782019 standard [46], samples were created using CATIA software (version V6 R22). The samples had rectangular prism shapes with dimensions of 4 mm × 10 mm × 100 mm. The 3D models were exported in STL format and loaded into Computer-Aided Manufacturing (CAM) software to prepare for the AM processes (for MED 610 and RGD720-Objet Studio, Stratasys Inc., Eden Prairie, MN, USA, version 9.2; for PEEK–3DGence Slicer (3DGence, Przyszowice, Poland, version 3.4); for PET-G HT100–PrusaSlicer (Prusa Research, Prague, Czech Republic, version 2.7.2)). The samples were fabricated in horizontal orientations, as shown in Figure 1.

Two methods were used to produce the samples. MED610 and RGD720 materials (Stratasys Inc., Eden Prairie, MN, USA) were printed using the Jetting Modeling Technology (MJT), while PEEK (3DGence, Przyszowice, Poland) and PET-G HT100 (Spectrum Filaments, Pecice, Poland) materials were printed using MEX technology. The MED610 and RGD720 samples were printed on a Stratasys Object CONNEX3 device (Stratasys, US and Canada). The printed structures for both materials included supports, which were removed using a pressure washer after the 3D printing process. The PEEK samples were printed on a 3DGence Industry F421 device (3DGence, Przyszowice, Poland), while the PET-G HT100 samples were printed on a 3D Prusa i3 MK3 device (Prusa Research, Prague, Czech Republic). The use of supports was required for 3D printing PET-G HT100, which were mechanically removed after 3D printing. The 3D printing parameters for each material are presented in Table 2. The printing parameters were chosen according to previous studies and manufacturer recommendations to achieve optimal material properties while ensuring repeatability in mechanical testing. For MEX-processed materials, factors such as nozzle temperature, bed temperature, and printing speed were adjusted to minimize defects such as warping and layer delamination, which can affect mechanical performance.

**Figure 2:**
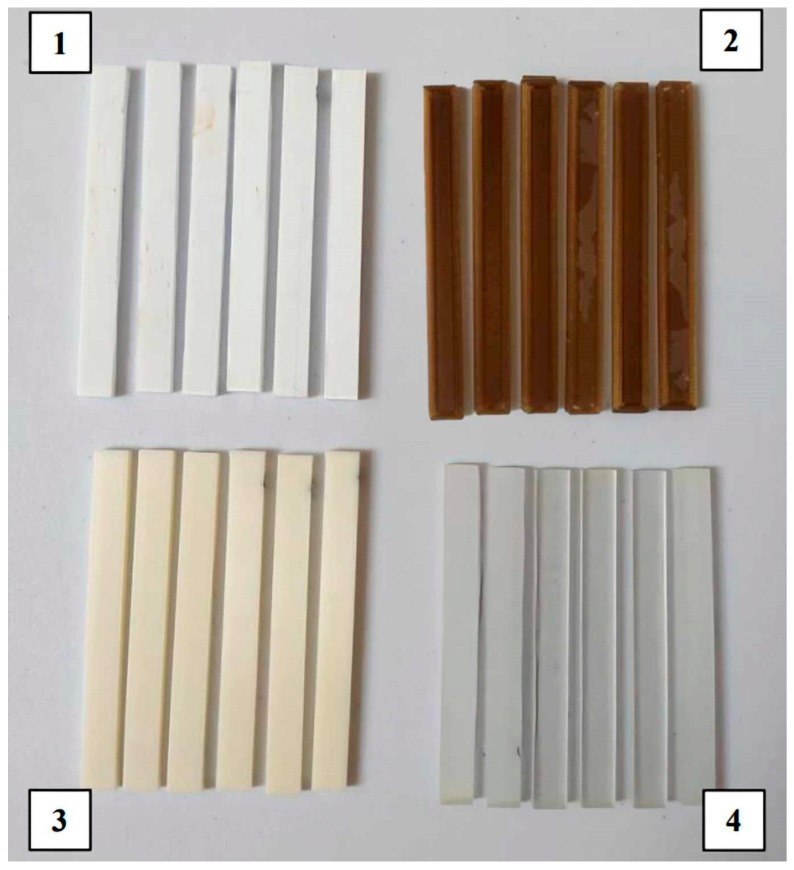
Printed samples: 1—PET-G HT100; 2—PEEK; 3—RGD720; and 4—MED610.

The printed and cleaned models of the samples are shown in Figure 2.

### 2.3. Steam Sterilization

The AM samples were subjected to a medical sterilization process using steam and a fractionated vacuum in an autoclave. To remove any microorganisms and contaminants from the samples’ surfaces, they were first subjected to washing and disinfection. Initially, the samples were washed with water, followed by the application of Medisept Viruton Bohr. In the next step, the samples were removed from the disinfectant solution, rinsed again, and then dried. To eradicate all microorganisms and perform sterilization, an E 12L BLACK IS YE-SON series sterilizer (IMPALL Rozwandowicz Bocheski Sp.K., Łódź, Poland) with a 12-liter chamber, classified as class B, was used.

**Figure 3:**
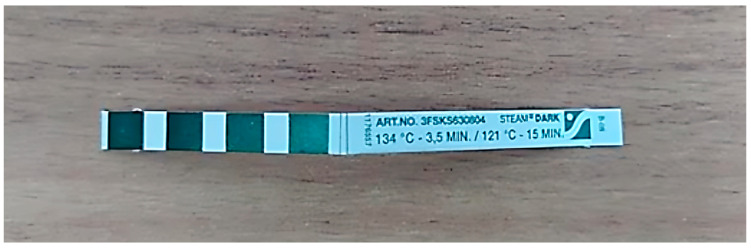
Indicator from the Bowie–Dick test after verification of sterilizer efficiency.

Subsequently, the functionality of the autoclave was verified. A Bowie–Dick test was conducted, which is required for sterilizers each day before they are used. The technical performance test, conducted after completing a test sterilization cycle, resulted in a color change in the chemical indicator of the paper (Figure 3). This indicated that the sterilization conditions in the equipment were appropriate.

**Figure 4:**
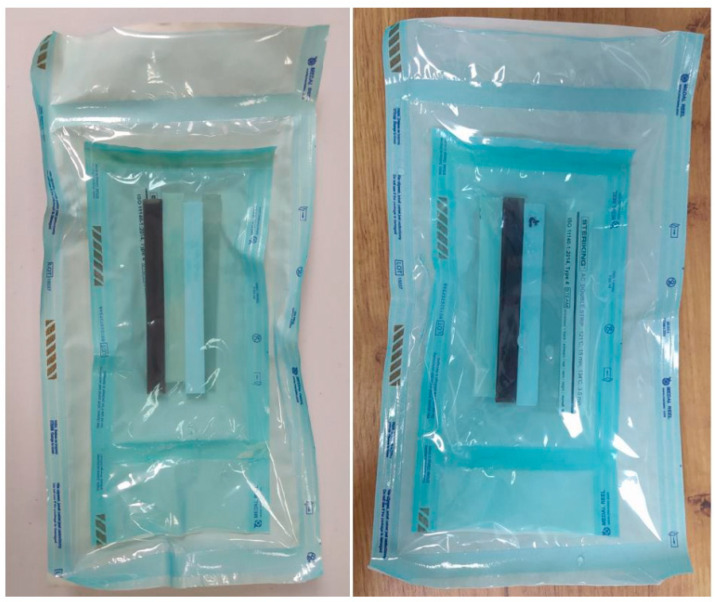
Packages after sterilization.

After drying the beams, ten sterilization packages were prepared, with two sterilization cycles assumed: 121 °C and 134 °C (five packages for each cycle). Each package contained one sample of each material and a type 4 indicator to verify the correctness of the sterilization process after completion. Additionally, the packages included a type 1 indicator to signal the end of the sterilization process. The samples, thus prepared, were subjected to the sterilization process. Upon completion of the process, the accuracy of the sterilization was verified. Figure 4 presents an example of the sterilization packages after two different temperature cycles.

In addition, after the sterilization process, a process report was printed on the device. The data from the report are presented in Table 3.

**Figure 5:**
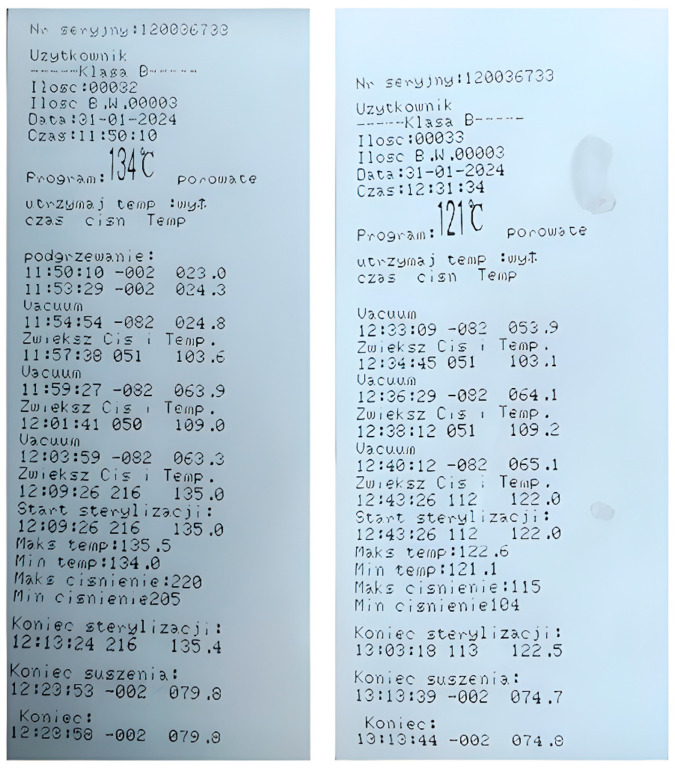
Reports on the sterilization process.

Table 3 contains selected parameters of the sterilization processes carried out. The course of saturated steam sterilization for porous products, as outlined in ISO 17665-1:2024 [47], is divided into six key phases. Figure 5 shows the process reports, which include information from each phase, including setting temperature, date, time, sterilization device (B), and maximum and minimum values of cycle parameters.

### 2.4. Testing Methods

**Figure 6:**
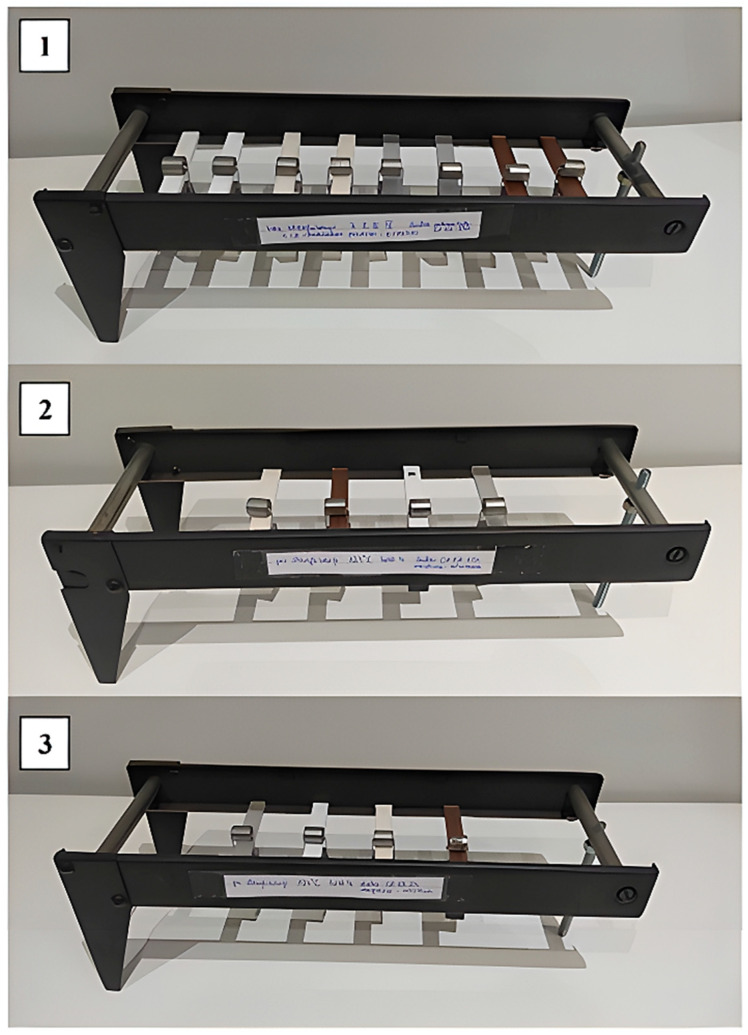
Samples placed at the bending test station (first day of the process): 1—without sterilization; 2—after sterilization at 121 °C; and 3—after sterilization at 134 °C.

To analyze the effect of steam sterilization on the selected mechanical properties of polymers, the samples were first subjected to a three-point bending test according to the ISO 178:2019 standard [46]. For this purpose, the samples were loaded, and each station was marked and arranged by supporting the specimens at two points on the stands. All samples were loaded with identical weights, resulting in a constant stress of 5 MPa on the materials. Three stands (shown in Figure 6) were set up, containing the printed beams as follows: 1—samples without sterilization; 2—samples after sterilization at 121 °C; and 3—samples after sterilization at 134 °C. The samples were subjected to loading on the stands for 4 months, which is approximately 2904 h. The environmental conditions for the process were as follows: temperature—20 ± 0.5 °C; humidity—50 ± 5%.

Using the MultiTest-dV 2.5 device (Mecmesin Ltd., Slinfold, UK), the maximum deflection of the samples (known as the deflection arrow), the compression force, and displacement over time during compression were measured. On the basis of the results obtained, the bending stresses were calculated using the following formula:

$$\sigma =\frac{3*F*L}{2*B*h^{2}}\;\left[\mathrm{Mpa}\right],$$

where

F—the force recorded during the sample deflection [N];

L—the span length between supports [mm];

b—the width of the sample [mm];

h—the thickness of the sample [mm].

Finally, Rockwell hardness testing was conducted using a Zwick/Roell device to assess the hardness of the polymer materials. For measurements, a spherical indenter with a diameter of 5 mm was used. For each sample, five measurements were recorded, and the two highest and two lowest values were excluded before performing the calculations. The distance between consecutive measurements was set to be three times the diameter of the indentation to avoid any mutual influence on the results.

## 3. Results and Discussion

### 3.1. Visual Analysis of Samples After Bench Testing 

The first step in the conducted research was the visual analysis of the specimens. Nominal samples were obtained directly after 3D printing, while samples studied included the samples after loading, the samples after sterilization at 121 °C and loading, and the samples after sterilization at 134 °C and loading. Analyzing the differences observed in the MED610 material samples (Table 4), a notable aspect after the three processes is the sample deflection. Minimal differences were observed in the beams that were also subjected to sterilization. Regarding the smoothness of the material surface and its color, no changes were detected. The observed characteristics of the MED610 material may suggest its reaction to steam sterilization. Apart from the noticeable deflection caused by loading, the polymer shows no significant deformations, and its color and visual structure remain unchanged.

Comparing the appearance of the PEEK samples (Table 5), a relatively small deflection was observed for each sample, regardless of the processes they underwent. Since there was no noticeable difference in deflection between the beams subjected to sterilization and those that were not, it can be inferred that PEEK exhibits resistance to sterilization processes and high temperatures. However, noticeable changes in the appearance of the beams were observed after sterilization. Samples subjected to steam sterilization were partially discolored and deformed on their larger surfaces. This could be attributed to the fact that moisture in the form of saturated steam affects the properties of PEEK, causing slight oxidation of the material on the surface, resulting in a change in appearance, despite its high melting temperature of approximately 343 °C.

The deflection of PET-G HT100 specimens (Table 6) is practically imperceptible. However, the noticeable differences between the beams before and after the decontamination process include the dulling of the samples after sterilization. Samples subjected to sterilization at 134 °C exhibit reduced smoothness and twisting. This could be due to surface oxidation of the polymer, microcrack formation, internal stresses that cause microscopic deformations, or contaminants in the water used for sterilization. However, these factors cannot be definitively determined based on the adopted analysis method, and identifying a specific cause would require additional research.

The RGD720 beam (Table 7), which was subjected to a four-month load, does not show any visual differences compared to the control samples, which did not undergo additional processes. The absence of visible deflection may confirm the high mechanical strength of this material. Minimal deflection, compared to the control samples, is only noticeable in the samples that underwent sterilization. This may suggest that sterilization affects the mechanical properties of the polymer, though this change is minimal.

### 3.2. Deflection 

**Figure 7:**
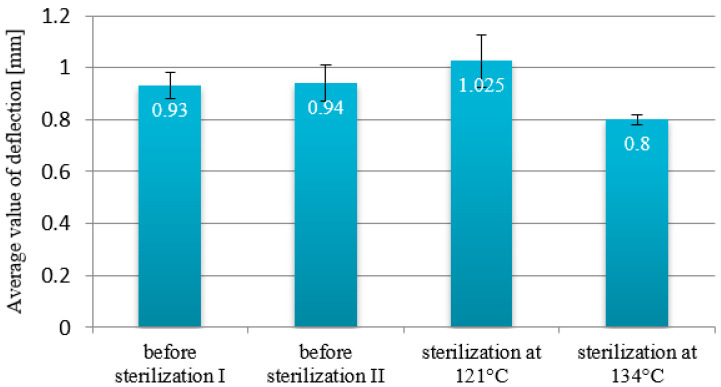
Deflection diagram for samples made from MED610 polymer.

The deflection values for the MED610 material samples that were not sterilized were 0.93 mm and 0.94 mm (Figure 7). The value of the samples that underwent the processes differs slightly: for the beams sterilized at 121 °C, it was 1.025 mm (an increase of 10%), while for the beams sterilized at 134 °C, it was 0.8 mm (a decrease of 14%). These findings suggest that sterilization has an impact on the deflection values of the material. Furthermore, the use of a temperature of 134 °C during sterilization, rather than the lower temperature, appears to strengthen the polymer.

**Figure 8:**
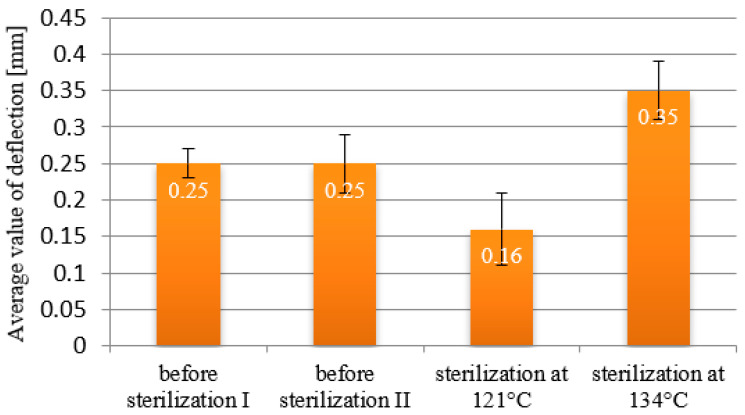
Deflection diagram for samples made from PEEK polymer.

The deflection values for the PEEK material samples are presented in Figure 8. Analyzing the deflection values of the samples, it can be observed that despite the absence of noticeable changes in the samples regarding the impact of sterilization on the degree of beam deformation, the measurements revealed differences. The deflection values for the samples before sterilization were 0.25 mm. Sterilization at 121 °C resulted in a 36% decrease in deflection value to 0.16 mm, while the same process performed at 134 °C resulted in a 40% increase in deflection value to 0.35 mm. The measurement results indicate that sterilization, depending on the set temperature, affects the material by increasing or decreasing its stiffness. However, this effect is minimal, as the differences in deflection values between sterilized and non-sterilized beams are generally around 0.1 mm.

**Figure 9:**
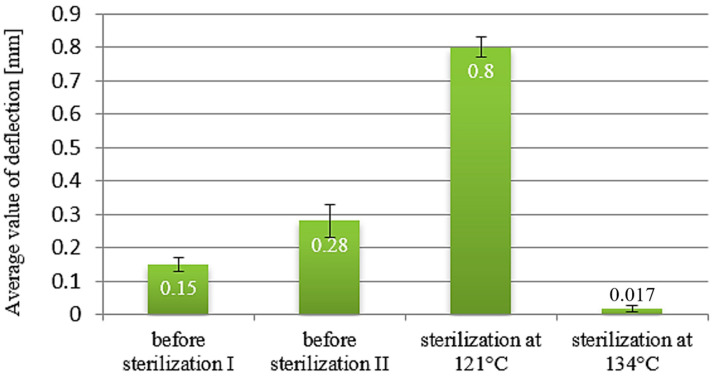
Deflection diagram for samples made from PET-G HT100 polymer.

The results of the deflection arrow measurements for beams made of PET-G HT100 material are illustrated in Figure 9. A comparison of the beam deflection values before and after sterilization indicates significant discrepancies for the PETG HT100 material. The values of the reference samples were 0.15 and 0.28 mm. The deflection value of the samples sterilized at 121 °C was four times higher, reaching 0.8 mm. This considerable difference suggests an increase in the flexibility of the material. For beams sterilized at 134 °C, a negligible deflection value of 0.017 mm was recorded (approximately a 10% decrease compared to reference values). This markedly different measurement result can be attributed to the beams that undergo deformation and twisting due to the applied processes. Therefore, this value does not accurately reflect the perpendicular deflection relative to the undeformed axis of the beams. 

**Figure 10:**
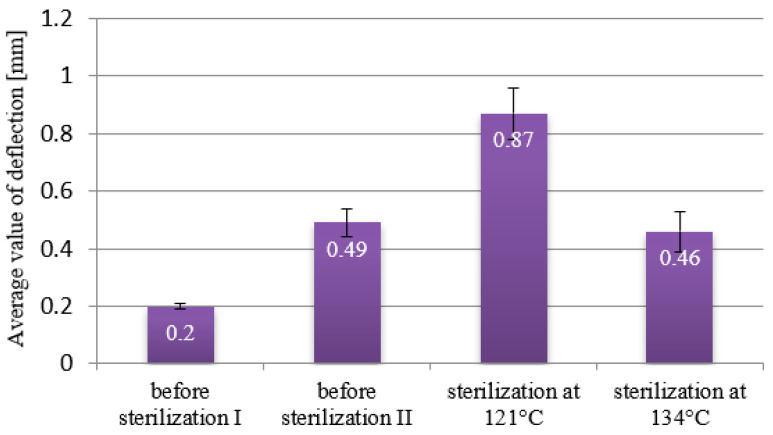
Deflection diagram for samples made from RDG720 polymer.

The data collected from the deflection arrow measurements of the RGD720 polymer are presented in Figure 10. Non-sterilized samples, which were subjected to four months of bending under constant load, showed deflection arrow values of 0.2 and 0.49 mm. For beams subjected to decontamination, a deflection of 0.87 mm was recorded. The differences in these values indicate a significant effect of sterilization on the mechanical properties of elements made from the RGD720 polymer material. The samples sterilized at 134 °C, as described for the polymer, experienced partial surface deformation. Although minimal, this deformation also influenced the result obtained, leading to significant differences in the measured deflection values of the beams subjected to the sterilization process. 

### 3.3. Three-Point Bending Test

**Figure 11:**
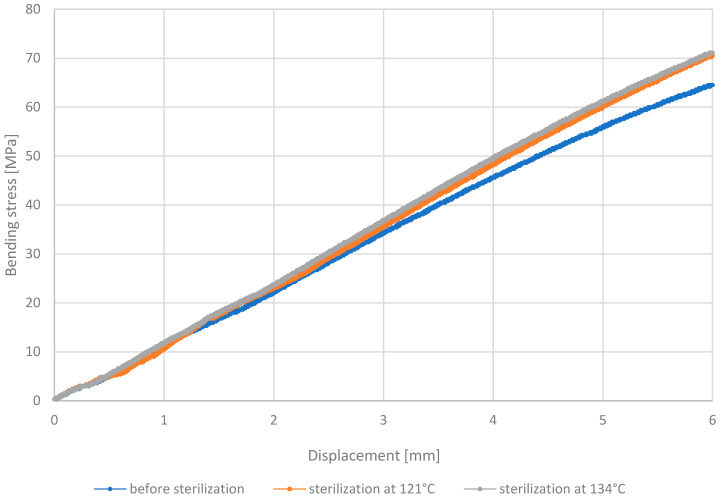
Representative stress–displacement relationship curves for MED610 material.

Based on the three-point bending test conducted on the MED610 material, it was determined that there is a linear relationship between the bending stress ratio and the applied displacement of the bending element for all types of samples tested: those without decontamination, those sterilized at 121 °C, and those sterilized at 134 °C. The results discussed are presented in Figure 11.

**Figure 12:**
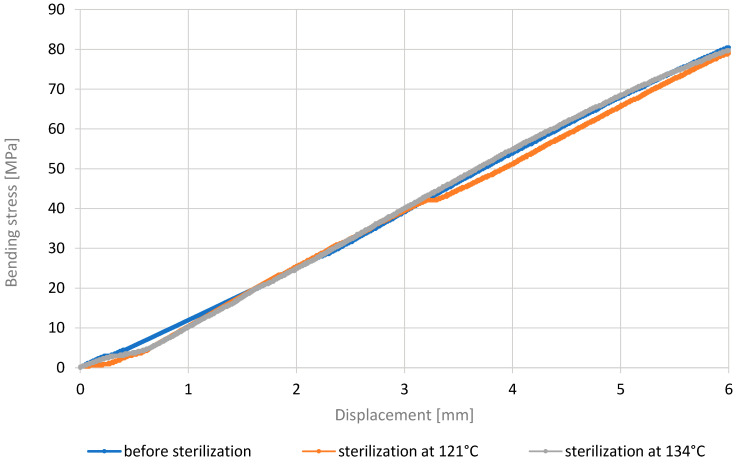
Representative stress–displacement relationship curves for PEEK material.

The increase in displacement is proportional to the amount of stress present. This uniform trend continues until a displacement length of 3 mm is reached for the bending element. Beyond this point, there is a slight deviation and an increase in stress values in the sterilized samples compared to those not subjected to this process. The curve illustrating changes in the non-sterilized sample shows lower bending stress values compared to the other samples, reaching approximately 6 MPa at a displacement of 6 mm. This indicates that samples subjected to decontamination exhibit greater resistance to bending of the material. Sterilization noticeably improves resistance to bending stresses, especially at 134 °C, where the stress values were even higher than at 121 °C. Such a strength enhancement of MED610 post-sterilization could be advantageous for medical products requiring increased mechanical resistance. The graph showing the stress–displacement relationship for the PEEK material is shown in Figure 12.

The curves depicting the stress–displacement relationship for the PEEK material, like for MED610, exhibit a linear increase in values. However, the stress values do not deviate significantly for the samples after the sterilization processes and show minor changes in stress resistance (up to approximately 3 MPa difference in the stress values). On the basis of this, it can be concluded that sterilization at both 121 °C and 134 °C has a negligible effect on the mechanical properties of PEEK. One probable reason and justification for these results could be that PEEK is printed at temperatures around 400 °C, making it resistant to high temperatures. Therefore, PEEK demonstrates resistance to sterilization processes. This property makes it suitable for applications where sterilization is necessary, along with the requirements for stability and high mechanical strength. The maximum bending stresses for all cases were approximately 80 MPa, which is higher compared to other studies [48,49] where the tested PEEK achieved maximum stresses in the range of 60 MPa.

**Figure 13:**
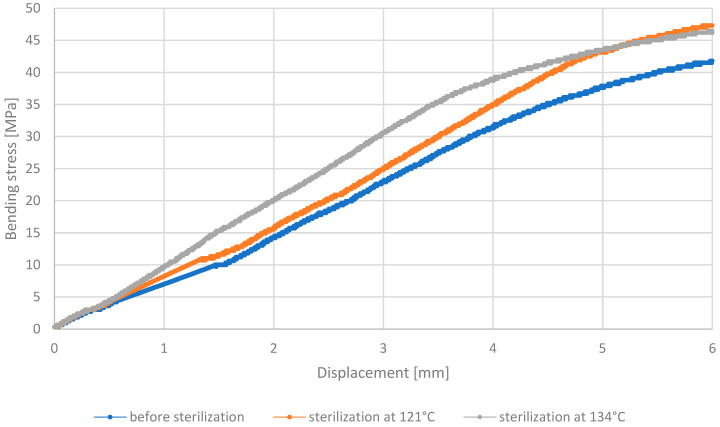
Representative stress–displacement relationship curves for RGD720 material.

In the three-point bending test for the RGD 720 material (Figure 13), significant differences were observed in the bending stress values for given displacements.

The stresses present in the beams that were not subjected to any sterilization were significantly lower than those in the sterilized beams. The specimen sterilized at 134 °C, compared to 121 °C, exhibited higher stress values between 1 and 5 mm of deflection; however, these stresses were later equalized. Despite differences in stress values among sterilized beams, the consistent observation was that for any given displacement length, the stresses in these beams did not exceed those in the non-sterilized beam. This indicates a significant effect of sterilization on the mechanical properties of the RGD720 material.

**Figure 14:**
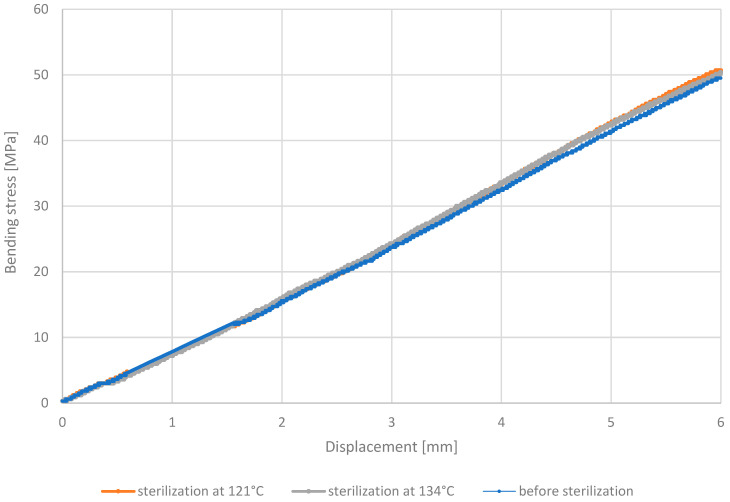
Representative stress–displacement relationship curves for PET-G HT100 material.

Measurement of samples made from the PET-G HT100 material in the three-point bending test revealed that regardless of whether the sample underwent sterilization (at 121 °C or 134 °C) or not, the bending stresses for given displacements were consistently similar for each beam. This suggests that the material is resistant to steam sterilization temperatures. This result confirms one of the characteristics of the material, which is its suitability for operation at elevated temperatures. The stress–displacement relationships for the PET-G HT100 material are illustrated in Figure 14.

### 3.4. Hardness

The outcomes of the Rockwell hardness tests indicate an increase in hardness for the materials MED610, PEEK, and RGD720 in samples subjected to steam sterilization, regardless of the specified temperature. Furthermore, for MED610, the highest hardness values were observed in samples sterilized at 121 °C. This may indicate a significant influence of saturated steam on the material at this temperature. PEEK achieved its highest hardness value after sterilization at 134 °C, with an increase in hardness of approximately 10 N/mm². The observed increase in PEEK hardness after sterilization at 134 °C can be attributed to several factors, such as reduced molecular mobility due to thermal exposure, increased cross-linking or secondary crystallization under high-temperature conditions, and the removal of moisture, which may have previously acted as a plasticizer. These findings are consistent with studies that indicate that controlled heat treatment can improve PEEK mechanical performance [50]. Similarly, the RGD720 polymer also showed higher measured values.

Only the hardness measurements of the PET-G HT100 polymer did not show significant changes in the results obtained between sterilized and non-sterilized samples. This suggests that steam sterilization does not have a significant impact on the hardness of the PET-G HT100 material and does not affect the likelihood of permanent deformation on its surface. All the results achieved are presented in Table 8.

### 3.5. Test Results Obtained

Table 9 summarizes all the results obtained for the four materials. MED610, PEEK, PET-G HT100, and RGD720 show clear differences in their mechanical properties when evaluated for deflection, flexural strength, and hardness both before and after sterilization at 121 °C and 134 °C.

The four materials, MED610, PEEK, PET-G HT100, and RGD720, show distinct differences in their mechanical properties, as observed in the table, when evaluated for deflection, bending strength, and hardness both before and after sterilization at 121 °C and 134 °C.

Before sterilization, MED610 shows the highest deflection (0.93–0.94 mm), indicating that it is the most flexible material, while PEEK has the lowest deflection (0.25 mm), suggesting that it is the most rigid. PET-G HT100 and RGD720 exhibit intermediate deflections, with PET-G HT100 ranging from 0.15 to 0.28 mm and RGD720 between 0.2 and 0.49 mm, indicating that these materials have moderate flexibility compared to MED610 and PEEK. In terms of bending strength, PEEK stands out with the highest value (81 MPa), followed by MED610 (70 MPa). PET-G HT100 and RGD720 show lower bending strengths (49 MPa and 44 MPa, respectively), indicating that they are less structurally robust. The hardness values are highest for MED610 (117.48 N/mm^2^), followed by PEEK (79.02 N/mm^2^), with PET-G HT100 and RGD720 showing significantly lower values (50.54 N/mm^2^ and 35.44 N/mm^2^, respectively), suggesting that MED610 and PEEK are harder and more resistant to surface indentation.

After sterilization at 121 °C, all materials exhibit an increase in deflection, MED610 showing the most significant change (from 0.93 mm to 1.025 mm), implying that it becomes more flexible at higher temperatures. PEEK, on the other hand, has the smallest increase in deflection (0.25 mm to 0.16 mm), demonstrating better stability in heat. PET-G HT100 and RGD720 also show increases in deflection, with RGD720 showing the largest increase (0.2 to 0.49 mm). In terms of bending strength, PEEK still maintains the highest value (79 MPa), with MED610 slightly decreasing to 64 MPa. PET-G HT100 and RGD720 experience smaller reductions in bending strength (51 MPa and 47 MPa, respectively), suggesting that while all materials lose some strength, PEEK retains the highest mechanical integrity. Regarding hardness, the hardness increases slightly to 134.02 N/mm^2^, while PEEK also shows a minor increase (83.74 N/mm^2^). PET-G HT100 and RGD720 show little change in hardness, with PET-G HT100 maintaining a hardness value of around 50 N/mm^2^, and RGD720 showing a small decrease to 50.38 N/mm^2^.

After exposure to 134 °C, the deflection values increase further for all materials, with MED610 experiencing the highest increase (to 0.8 mm), suggesting a significant reduction in rigidity at this higher sterilization temperature. PEEK deflection remains relatively low (0.16 mm to 0.87 mm), demonstrating its superior resistance to deformation under high heat. PET-G HT100 and RGD720 both show moderate increases, with the deflection of PET-G HT100 increasing to 0.35 mm. Regarding the bending strength, PEEK retains the highest value (80 MPa), followed by MED610 (71 MPa), which remains relatively strong despite the increase in temperature. PET-G HT100 and RGD720 show further reductions in strength, with RGD720 dropping to 46 MPa. In terms of hardness, the hardness decreases slightly to 116.40 N/mm^2^, while PEEK maintains its hardness (84.86 N/mm^2^). PET-G HT100 and RGD720 exhibit stable hardness after exposure to 134 °C, with PET-G HT100 at 49.78 N/mm^2^ and RGD720 at 45.44 N/mm^2^.

In conclusion, PEEK demonstrates superior performance in terms of rigidity, bending strength, and stability at high temperatures, making it the most reliable material in this comparison. MED610, while more flexible, retains a relatively high bending strength and hardness, but shows more pronounced changes in deflection after sterilization. PET-G HT100 offers moderate performance in all properties but is notably stable in terms of deflection across temperature changes. RGD720, on the other hand, shows the lowest performance in most parameters, particularly in bending strength and hardness, suggesting that it may not be suitable for applications that demand high structural integrity or resistance to deformation.

## 4. Conclusions

The research aimed to assess the impact of steam sterilization on the mechanical properties of selected polymer biomaterials used in medical engineering. The study focused on four types of materials: MED610, PEEK, PET-G HT100 and RGD720, from which samples were AM. These samples underwent sterilization processes at temperatures of 121 °C and 134 °C, followed by long-term loading to evaluate changes in their mechanical properties. Based on research and analyses, which are integral parts of this study, the following conclusions can be formulated regarding the effect of the medical sterilization process on the mechanical properties of selected polymer biomaterials.

- The steam sterilization process significantly affects the mechanical properties of polymer biomaterials such as MED610, PEEK, PETG HT100, and RGD720. Strength tests indicate varied reactions of these materials to the sterilization process, which is crucial for their further application in medicine.
- Creep tests and deflection measurements showed that steam sterilization leads to higher deflection values in sterilized beams. These results suggest that the temperature of the sterilization process is a significant factor that influences the mechanical properties of these materials.
- Analysis of the outcomes of the three-point bending test revealed that PETG HT100 and PEEK materials, characterized by high 3D printing temperatures, exhibit substantial resistance to the sterilization process, showing no significant differences in stress values after sterilization. On the contrary, the other materials examined showed increased bending stress values after sterilization, suggesting that this process may enhance their bending strength. The resistance of PETG HT100 and PEEK to sterilization suggests that materials with similar properties could be further developed to ensure high mechanical stability in medical applications. Furthermore, the observed increased bending strength in some materials after sterilization suggests that, in some cases, the sterilization process may enhance certain material properties, which could be leveraged in the design of materials for specific medical applications.
- The hardness test indicated that most materials, except PETG HT100, achieve higher hardness values after steam sterilization. The increased hardness of the material suggests a reduction in its susceptibility to permanent deformation under mechanical forces. PETG HT100 did not show significant differences in hardness values after sterilization, indicating its mechanical stability at elevated temperatures. The increase in hardness in many materials after sterilization suggests that engineers could design materials with improved resistance to permanent deformation, making them more durable for use in medical devices. The stability of PETG HT100 also highlights the potential for the development of materials that retain their mechanical properties under sterilization conditions, a critical factor for their use in the medical field.

The research findings indicate that the medical sterilization process should be considered when designing and manufacturing polymer materials used in medicine. Differences in material behavior after sterilization may have significant implications for their clinical application, particularly in the context of the creation of implants and other medical devices. This study provides a foundation for further analysis of the effect of sterilization on the mechanical properties of biomaterials. The outcomes may contribute to the development of new polymer materials and AM technologies that are better suited for sterilization and consequently more appropriate for medical applications. The results indicate that PEEK and PET-G HT100 are highly resistant to sterilization-induced mechanical degradation, which makes them suitable candidates for applications that require repeated sterilization, such as surgical instruments and reusable medical components. On the contrary, materials such as MED610 and RGD720 may be more appropriate for single-use applications where post-sterilization mechanical reinforcement is beneficial. Continued work on optimizing the mechanical properties of polymers could lead to the more frequent use of these materials in the production of personalized medical implants customized to individual patient needs. Furthermore, by addressing the specific weaknesses identified in the materials studied, smart design solutions could be applied to overcome these limitations, ensuring that the materials used for medical devices and implants offer enhanced performance and longevity. Through the continued integration of advanced material science and sterilization processes, the next generation of polymer biomaterials could be better equipped to meet the evolving demands of modern medical engineering.

## Figures and Tables

**Table 1:** Properties of polymeric materials [40,41,42,43,44,45].

	MED610	PEEK	PET-G HT100	RGD720
Tensile Strength [MPa]	50–65	110	43	50–65
Bending Strength [MPa]	75–110	130	52	80–110
Young’s Modulus [MPa]	2000–3000	4100	1575	2300–2700

**Table 2:** Three-dimensional printing settings for the different materials.

	Layer Thickness [mm]	Printing Speed [mm/s]	Nozzle Temperature [°C]	Bed Temperature [°C]	Chamber Temperature [°C]	Type of Infill	Number of Contours	Infill [%]
MED610	high quality	-	-	-	-	standard	-	100
PEEK	0.15	50	420	100	110	parallel lines	5	100
PET-G HT100	0.2	45	250	100	-	parallel lines	2	100
RGD720	high quality	-	-	-	-	standard	-	100

**Table 3:** Data from the sterilization process report.

	Max Temperature [°C]	Min Temperature [°C]	Max Pressure [MPa]	Min Pressure [MPa]	Class	Program
Sterilization at 121 °C	122.6	121.1	115	104	B	121 °C, porous
Sterilization w 134 °C	135.5	124.0	220	205	B	134 °C, porous

**Table 4:** Representative results of visual analysis of MED610 printed shapes after various processes.

Ref.	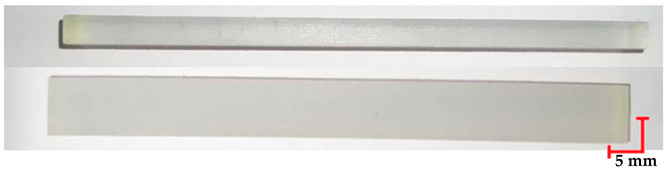
Sample after bending test	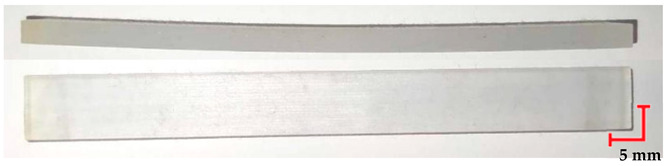
Sample after sterilization at temperature 121 °C and bending test	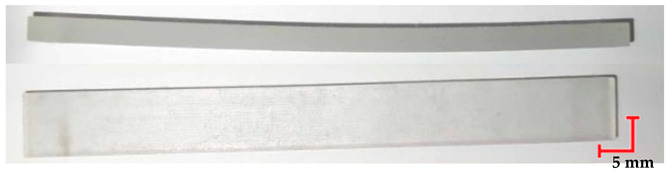
Sample after sterilization at temperature 134 °C and bending test	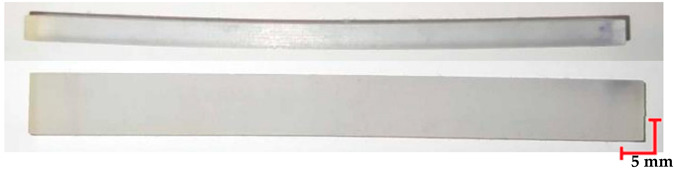

**Table 5:** Representative results of visual analysis of PEEK printed shapes after various processes.

Ref.	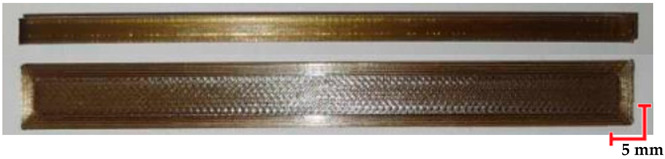
Sample after bending test	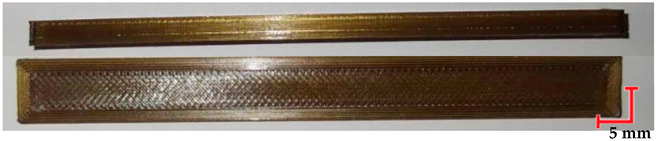
Sample after sterilization at temperature 121 °C and bending test	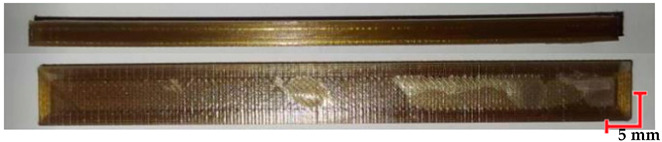
Sample after sterilization at temperature 134 °C and bending test	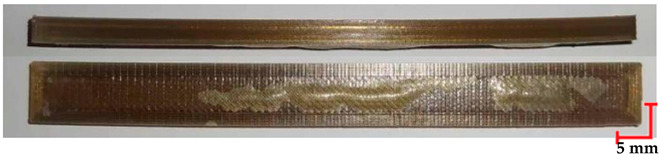

**Table 6:** Representative results of visual analysis of PET-G HT100 printed shapes after various processes.

Ref.	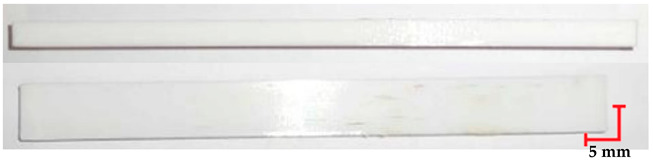
Sample after bending test	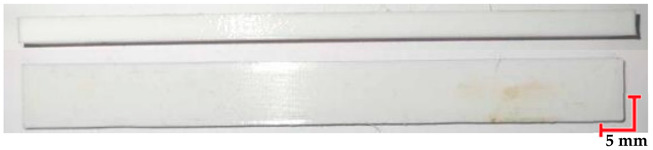
Sample after sterilization at temperature 121 °C and bending test	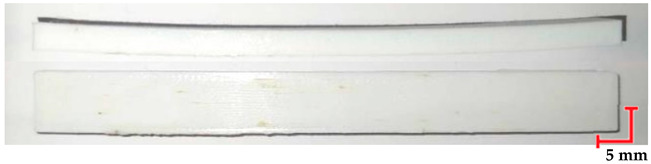
Sample after sterilization at temperature 134 °C and bending test	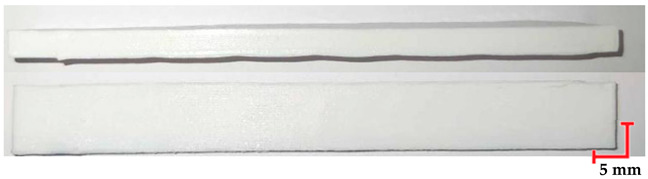

**Table 7:** Representative results of visual analysis of RGD720 printed shapes after various processes.

Ref.	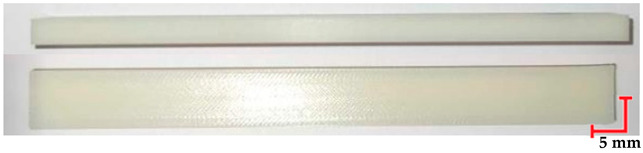
Sample after bending test	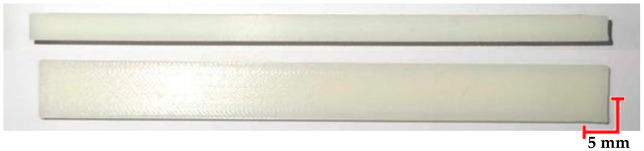
Sample after sterilization at temperature. 121 °C and bending test	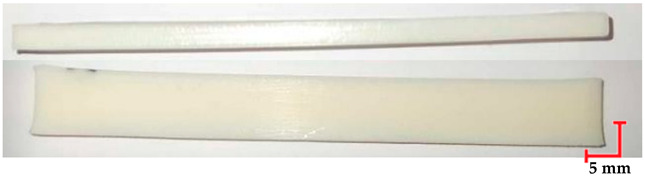
Sample after sterilization at temperature 134 °C and bending test	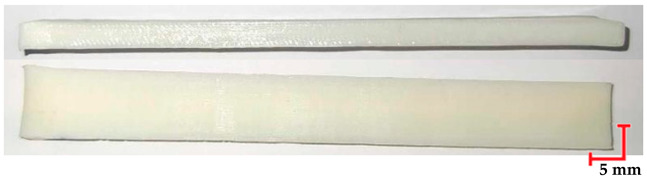

**Table 8:** Rockwell hardness test results for individual samples.

	MED610 [N/mm^2^]	PEEK [N/mm^2^]	PET-G HT100 [N/mm^2^]	RGD720 [N/mm^2^]
Test load [N]	358	358	132	132
Without Sterilization	117.48 ± 8.56	79.02 ± 0.81	50.54 ± 3.31	35.44 ± 1.05
After Sterilization at 121 °C	134.02 ± 1.30	83.74 ± 12.59	49.98 ± 7.56	50.38 ± 9.05
After Sterilization at 134 °C	116.40 ± 10.97	84.86 ± 11.06	49.78 ± 6.69	45.44 ± 6.68

**Table 9:** Results of all tests.

Material	MED610	PEEK	PET-G HT100	RGD720
Deflection Before Sterilization [mm]	0.93–0.94 ± 0.07	0.25 ± 0.04	0.15–0.28 ± 0.02	0.2–0.49 ± 0.05
Deflection After 121 °C [mm]	1.025 ± 0.01	0.16 ± 0.05	0.8 ± 0.03	0.87 ± 0.09
Deflection After 134 °C [mm]	0.8 ± 0.02	0.35 ± 0.04	0.017 ± 0.01	0.46 ± 0.07
Bending Strength Before Sterilization [MPa]	70	81	49	44
Bending Strength After 121 °C [MPa]	64	79	51	47
Bending Strength After 134 °C [MPa]	71	80	50	46
Hardness Before Sterilization [N/mm^2^]	117.48 ± 8.56	79.02 ± 0.81	50.54 ± 3.31	35.44 ± 1.05
Hardness After 121 °C [N/mm^2^]	134.02 ± 1.30	83.74 ± 12.59	49.98 ± 7.56	50.38 ± 9.05
Hardness After 134 °C [N/mm^2^]	116.40 ± 10.97	84.86 ± 11.06	49.78 ± 6.69	45.44 ± 6.68

## Data Availability

The original contributions presented in the study are included in the article; further inquiries can be directed to the corresponding author.
